# Supplementary figures and images for: Development of an Artificial Neural Network for the Detection of Supporting Hindlimb Lameness: A Pilot Study in Working Dogs
Source: Animals (Basel). 2022 Jul 8;12(14):1755. doi: 10.3390/ani12141755 (PMC9311578; doi:10.3390/ani12141755)

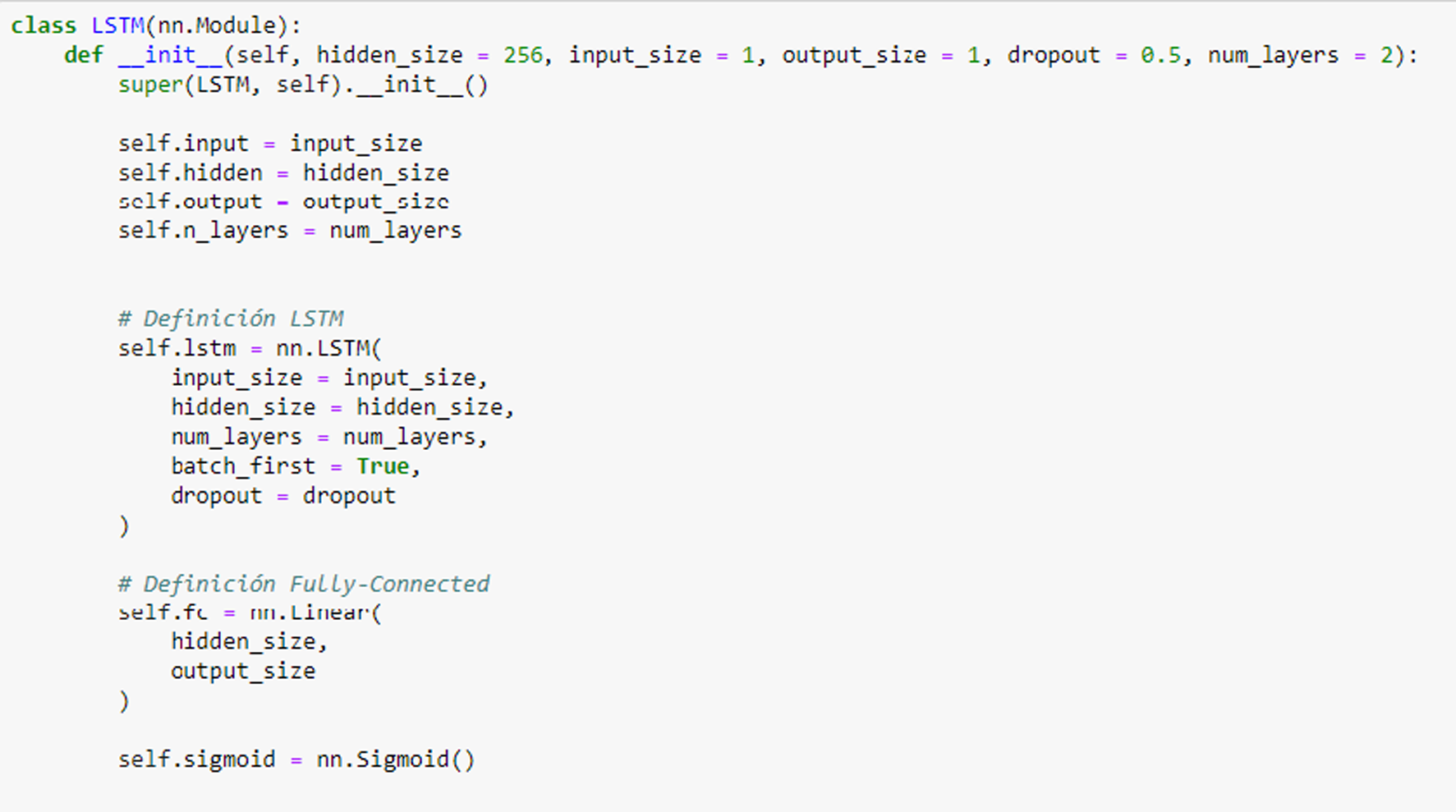

Supplement: Supplementary file 1 [file animals-12-01755-s001.zip › animals-1761127-supplementary/Figure S1.tiff]

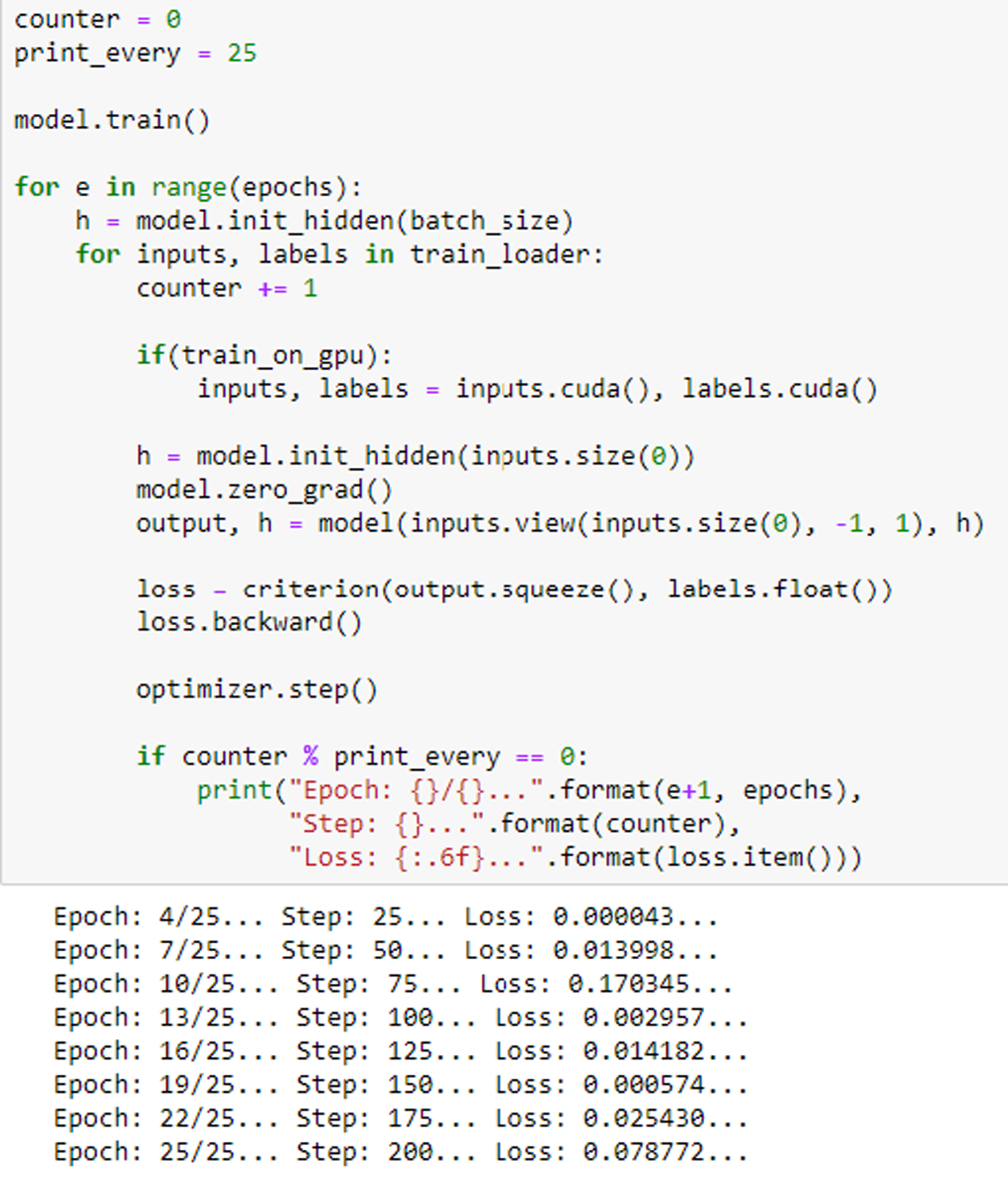

Supplement: Supplementary file 1 [file animals-12-01755-s001.zip › animals-1761127-supplementary/Figure S2.tiff]

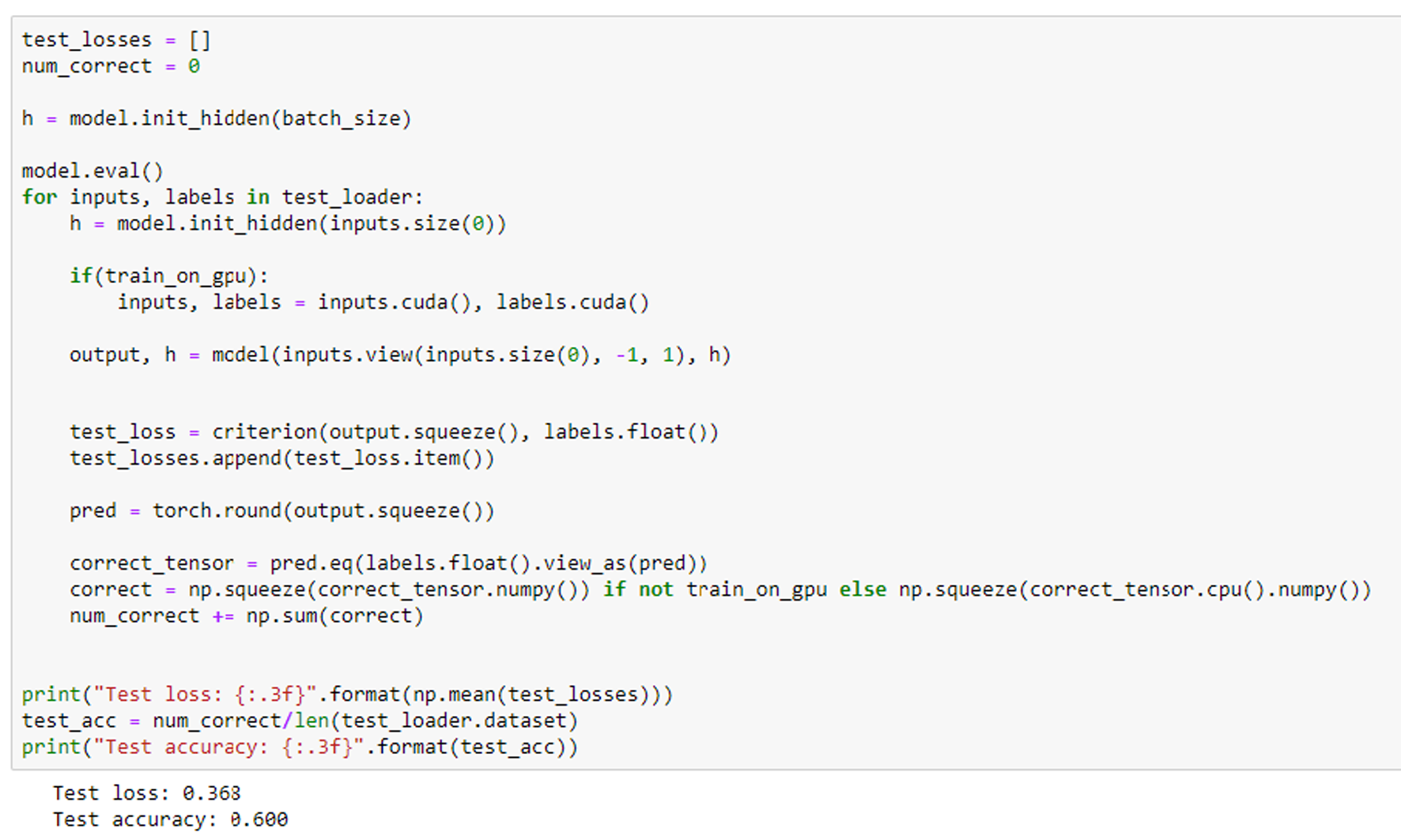

Supplement: Supplementary file 1 [file animals-12-01755-s001.zip › animals-1761127-supplementary/Figure S3.tiff]

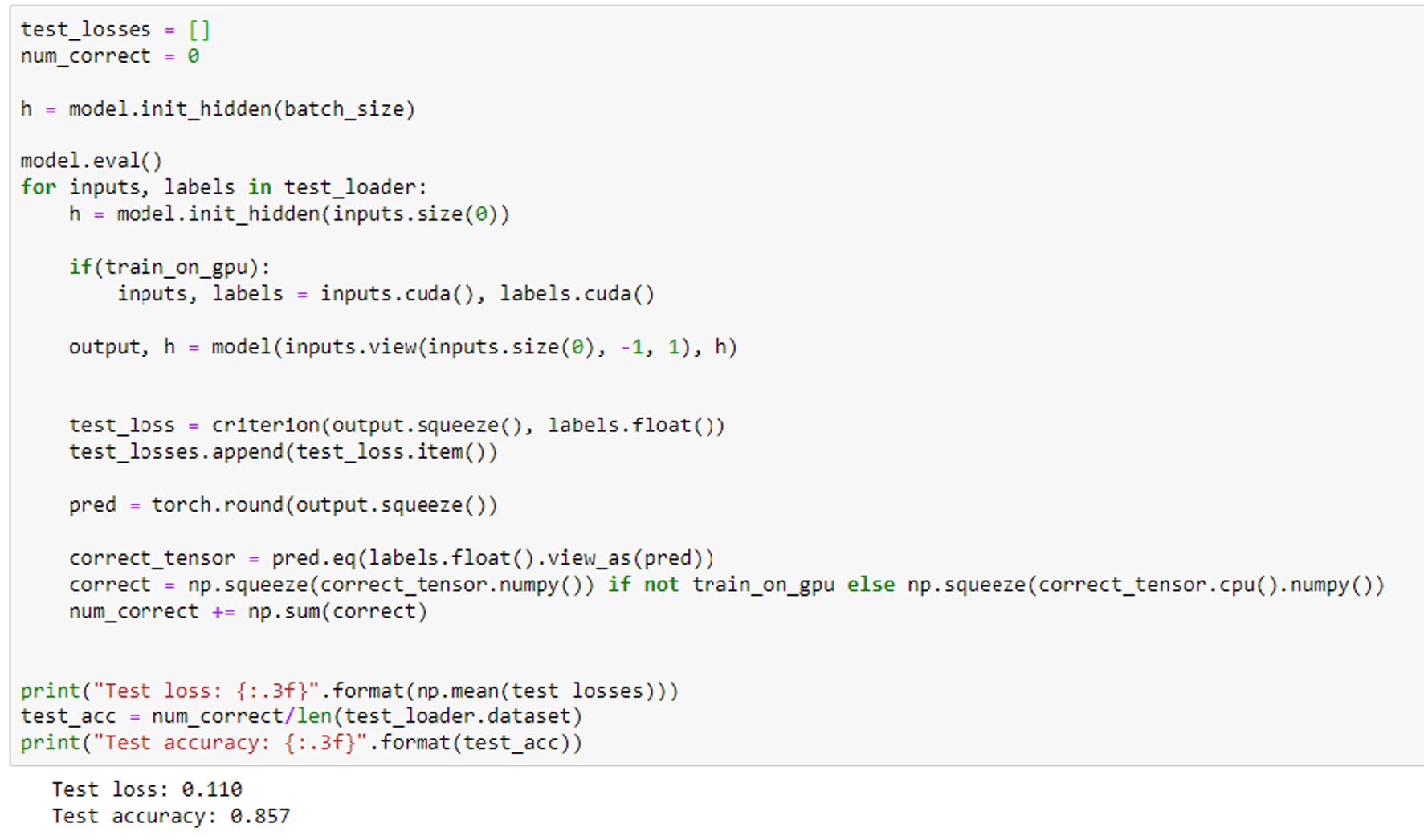

Supplement: Supplementary file 1 [file animals-12-01755-s001.zip › animals-1761127-supplementary/Figure S4.tiff]
